# Tissue Architecture Influences the Biological Effectiveness of Boron Neutron Capture Therapy in In Vitro/In Silico Three-Dimensional Self-Assembly Cell Models of Pancreatic Cancers

**DOI:** 10.3390/cancers13164058

**Published:** 2021-08-12

**Authors:** Lin-Sheng Yu, Megha Jhunjhunwala, Shiao-Ya Hong, Lin-Yen Yu, Wey-Ran Lin, Chi-Shuo Chen

**Affiliations:** 1 Department of Biomedical Engineering and Environmental Sciences, National Tsing Hua University, Hsinchu 30013, Taiwan; op7532000@gmail.com (L.-S.Y.); jhunjhunwala16@gmail.com (M.J.); linyen0701@gmail.com (L.-Y.Y.); 2 Medical Research Center, Cardinal Tien Hospital, New Taipei City 23148, Taiwan; megha.shaline9@msn.com; 3 Department of Gastroenterology & Hepatology, Linkou Chang Gung Memorial Hospital, Taoyuan 33305, Taiwan; 4 Department of Medicine, Chang Gung University, Taoyuan 33302, Taiwan

**Keywords:** BNCT, 3D tumoroid, tumor microenvironment, pancreatic cancer, in silico simulation, cellular potts model

## Abstract

**Simple Summary:**

Boron neutron capture therapy (BNCT) is becoming one of the most promising radiotherapies for aggressive cancers, but the detailed cellular mechanisms of BNCT remain largely underexplored. Solid tumors are composed of heterogeneous cell populations, which create a 3-dimensional complicated microenvironment for tumor progression. To recapture the influences of the microenvironment on BNCT efficacy, we applied a self-assembly 3D cell culture system with two different types of pancreatic cancer cells. In contrast to previous findings with γ-ray exposure, we found that the 3D architecture of pancreatic tumor can facilitate the susceptibility of cancer cells to BNCT, as compared to 2D tissue structure; a computer simulation model was established to further confirm this unexpected finding. These outcomes can contribute to better understanding of the radiobiology of BNCT, and the developed models may facilitate the recent development in personalized radiotherapy.

**Abstract:**

Pancreatic cancer is a leading cause of cancer death, and boron neutron capture therapy (BNCT) is one of the promising radiotherapy techniques for patients with pancreatic cancer. In this study, we evaluated the biological effectiveness of BNCT at multicellular levels using in vitro and in silico models. To recapture the phenotypic characteristic of pancreatic tumors, we developed a cell self-assembly approach with human pancreatic cancer cells Panc-1 and BxPC-3 cocultured with MRC-5 fibroblasts. On substrate with physiological stiffness, tumor cells self-assembled into 3D spheroids, and the cocultured fibroblasts further facilitated the assembly process, which recapture the influence of tumor stroma. Interestingly, after 1.2 MW neutron irradiation, lower survival rates and higher apoptosis (increasing by 4-fold for Panc-1 and 1.5-fold for BxPC-3) were observed in 3D spheroids, instead of in 2D monolayers. The unexpected low tolerance of 3D spheroids to BNCT highlights the unique characteristics of BNCT over conventional radiotherapy. The uptake of boron-containing compound boronophenylalanine (BPA) and the alteration of E-cadherin can partially contribute to the observed susceptibility. In addition to biological effects, the probability of induced α-particle exposure correlated to the multicellular organization was speculated to affect the cellular responses to BNCT. A Monte Carlo (MC) simulation was also established to further interpret the observed survival. Intracellular boron distribution in the multicellular structure and related treatment resistance were reconstructed in silico. Simulation results demonstrated that the physical architecture is one of the essential factors for biological effectiveness in BNCT, which supports our in vitro findings. In summary, we developed in vitro and in silico self-assembly 3D models to evaluate the effectiveness of BNCT on pancreatic tumors. Considering the easy-access of this 3D cell-assembly platform, this study may not only contribute to the current understanding of BNCT but is also expected to be applied to evaluate the BNCT efficacy for individualized treatment plans in the future.

## 1. Introduction

Pancreatic cancer is the third leading cause of death from cancer in the United States [1]. Pancreatic ductal adenocarcinoma, an exocrine tumor that starts in the ducts of the pancreas, accounts for approximately 85% of the incidences of pancreatic cancer [2]. Symptoms are usually not present until the tumor reaches the metastatic stage [3]. The treatment of pancreatic cancer remains challenging, and the average 5-year survival rate for people with pancreatic cancer is only 10% [4]. More than 50% of patients with cancer receive radiation therapy. Typically, radiation therapy can be applied with chemotherapy or combined with surgery to attempt tumor removal [5,6,7]. An emerging topic in radiation oncology is the development of high-energy particles for tumor treatment [8]. Boron neutron capture therapy (BNCT), which uses the limited path length (5–9 µm) of high linear energy transfer particles and recoiling lithium, generated when thermal neutrons are incident on boron-10, can selectively destroy tumor cells with minimal injury to non-cancer tissue [9]. Recently, with the development of neutron sources and boron agents, BNCT is becoming one of the most promising radiotherapies for aggressive cancers. For instance, a significant increase of survival rate of patients with glioblastoma was reported using accelerator-based BNCT [10]. Although the promising efficacy of BNCT for recurrent malignant gliomas and head and neck cancers has been reported in clinical studies [11], the potency of BNCT for pancreatic cancer treatment has not been fully explored.

The importance of the tumor microenvironment in regulating the biological effects of tumors has been demonstrated in various studies [12,13]. Solid tumors are composed of heterogeneous cell populations, including cancer cells, stroma cells, and immune cells [12,14]. For example, during the progression of desmoplasia in pancreatic cancer, a dense fibrous stroma composed of type I collagen and hyaluronic acid is deposited by the surrounding fibroblasts; the resulting hypovascular and hypoxic desmoplastic microenvironment shields the tumor from the chemotherapy agents [2]. In addition to altering the ECM matrix, cancer-associated fibroblasts also contribute to radioresistance in tumor cells by mediating their secretion factors and contact signals [15]. Compared with other high-energy particle treatments, such as proton- and electron-based approaches, BNCT generates radiation only in drug-uptaking cells [16]. Thus, the interstitial diffusion of boron-containing agents is hindered by the tumor microenvironment; this is a key factor affecting the therapeutic effect of BNCT.

Relative biological effectiveness (RBE), indicating the ratio of biological damage to absorbed radiation energy, is broadly used to estimate the efficacy of radiation treatment [8,9]. To compare the efficacy of ionizing radiation, biological models have been developed to estimate RBE, such as tumor size, cell viability, and reactive oxygen species level [17]. Animal models and xenografts have been widely adapted as preclinical approaches to evaluate the RBE [18]. However, the average rate of translation from animal model to clinical cancer patients is less than 8% [19]. Recently, organoids and tumoroids, which are in vitro three-dimensional (3D) multicellular structures, were demonstrated to capture the cellular characteristics of in vivo organs and tumors [20]. In contrast to the conventional two-dimensional (2D) in vitro culture wherein cells grow in a monolayer, the 3D architecture of tumoroid provides abundant cell-cell contacts and a rich microenvironment, showing great potential to explore therapeutic resistance [21,22]. Furthermore, in silico models are developed to offer comprehensive insights into each individual parameter that affects the cellular physiology [23,24]; by appending cell characteristics, environmental factors and biochemical signals, various cell responses can be applied from multiple parametric dimensions, such as tumor growth and cell death, for estimating the efficacy of treatment [25,26].

In this study, we aimed to evaluate the compound biological effectiveness of BNCT using the self-assembly 3D cell culture system. In addition to using molecular and cellular tools to study the radiation responses of pancreatic cancer in vitro, we adopted cellular Potts model (CPM) to simulate biological responses in silico with different physiological parameters, such as boron uptake and multicellular organization. We expect the achievements of the study to contribute towards a better understanding of BNCT in cancer therapy at the multicellular organization level.

## 2. Methods and Material

### 2.1. Cell Culture

Panc-1 and BxPC-3 human pancreatic cancer cell lines and the MRC-5 human lung fibroblast cell line were obtained from the Bioresource Collection and Research Center. Panc-1 cells were cultured in high-glucose Dulbecco’s modified Eagle medium (DMEM, Thermo Fisher Scientific, Carlsbad, CA, USA). BxPC-3 cells were cultured in RPMI-1640 with L-glutamine (Corning Inc., Manassas, VA, USA). All culture mediums were supplemented with 10% inactive fetal bovine serum (Thermo Fisher Scientific, Carlsbad, CA, USA) and 100 U/mL penicillin–streptomycin (Thermo Fisher Scientific, Carlsbad, CA, USA) and cultured at 37 °C in a humidified atmosphere containing 5% CO_2_. MRC-5 cells were cultured in Eagle’s minimum essential medium with 1.5 g/L sodium bicarbonate, nonessential amino acids, L-glutamine, and sodium pyruvate (Corning Inc., Manassas, VA, USA).

### 2.2. 3D Tumoroid Formation and Morphology

We produced a polyacrylamide (PA) hydrogel by following the protocol in [27] and coating the substance with collagen type I (Corning Inc., Manassas, VA, USA). Subsequently, the Panc-1 and BxPC-3 cells were seeded alone or with MRC-5 on PA hydrogel inside 24-well culture plates. The seeding density for each well was 5 × 10^4^, and the ratio of cancer cells to fibroblasts was 2:3. The culture medium was changed every 2 days. Until day 7, we observed and captured images using Nikon ECLIPSE Ti microscopy and a Hamamatsu ORCA-flash 4.0 camera. Images were later analyzed using ImageJ 1.53j software.

### 2.3. Immunofluorescence and Confocal Microscopy

Cell samples were fixed for 30 min with 4% paraformaldehyde, permeabilized with phosphate-buffered saline containing 0.5% Triton X-100, and then blocked by 2% bovine serum albumin in phosphate-buffered saline containing 0.5% Tween for 30 min. The samples were incubated overnight at 4 °C with the corresponding primary anti-E-cadherin 1:300 (GTX629694) and anti-γH2AX 1:200 (GTX628789) then stained with secondary antibodies conjugated with Alexa 488 and 594(Jackson ImmunoResearch Inc, West Grove, PA, USA). The cell nuclei were stained using hoechst and Sytox Green and visualized using an Olympus IX 81 Confocal Laser Scanning Microscope. The 3D tumoroid was rendered transparent by using RapiClear 1.49 (SUNJin Lab, Hsinchu, Taiwan) before images were captured. Images were later analyzed using ImageJ 1.53j software.

### 2.4. Western Blot and Flow Cytometry

All cells were collected on day 7 and lysed by Trident RIPA lysis buffer (GeneTex Inc., Irvine, CA, USA) plus Halt™ protease inhibitor cocktail, EDTA-Free (100X) (Thermo Fisher Scientific, Carlsbad, CA, USA) for 30 min. Subsequently, the samples were mixed with 4X Laemmli sample buffer (Bio-rad Laboratories, Inc., Hercules, CA, USA) containing 2.5% β-mercaptoethanol (Sigma-Aldrich, Taufkirchen, Germany) and heated at 95 °C for 5 min. Next, cell samples were loaded onto 10% PA gels for electrophoresis. Proteins were transferred to poly (vinylidene fluoride) membranes blocked with 5% dry milk, Tris buffer saline, and 0.2% Tween and incubated with primary antibodies anti-E-cadherin 1:2000 (GTX629694) and anti-actin 1:10,000 (GTX629630) overnight at 4 °C and stained with horseradish-peroxidase-coupled secondary antibodies goat-antimouse 1:5000 (Jackson ImmunoResearch Inc, West Grove, PA, USA) for 1 h at room temperature. Bands were revealed using Trident femto Western HRP substrate (GTX14698) and detected using Syngene’s G:Box. Quantification of band intensity was performed using ImageJ 1.53j software.

Twenty four hours after irradiation, the cells were separated by Accutase (Thermo Fisher Scientific, Carlsbad, CA, USA) and stained with viability assay marker (calcein AM and PI) and apoptosis assay marker (Annexin V and PI). Subsequently, the samples were analysed using BD FACSCanto^TM^ Flow Cytometry. All results were processed by FlowJo 7.6 software.

### 2.5. BNCT and Boronophenylalanine Uptake

Cells were cultured for 7 days and then treated with 25 ug/mL boronophenylalanine (BPA) for 4 h. Subsequently, we removed BPA before BNCT irradiation and transported the sample from the incubator to THOR (Tsing Hua Open-Pool Reactor) which belongs to the National Tsing Hua University in Taiwan. The beam aperture was 14 cm in diameter. The irradiation condition was 1.2 MW for 28 min while the source intensities of thermal, epithermal and fast neutrons at the 14 cm diameter beam aperture were 6 × 10^7^, 8.2 × 10^8^ and 6.4 × 10^7^ cm^−2^ s^−1^, respectively [2,4]. The culture medium was removed, and cells were detached using trypsin. The cell samples were treated with 65% nitric acid overnight at 4° C. Analysis of the samples was conducted using an Agilent 7500ce inductively coupled plasma mass spectrometer (ICP-MS).

### 2.6. In Silico Simulation

A Monte Carlo simulation model is established using CompuCell 3D platform [24]. Briefly, each cell area is defined at 25 pixels with contact energy at 8 per unit contact area for each cell pair to represent the intercellular interactions. The simulation progresses by trying to minimize the overall system energy. In this study, the alpha particle propagation constants are varied between 0.1 and 0.2 pixel^2^/MCS with decay constant at 0.01/MCS to recapture the interaction in different materials.

### 2.7. Statistical Analysis

Mean ± standard deviation (SD) of at least three independent experiments were calculated. To test statistical significance, Student’s *t*-test was done using GraphPad 8.0. Results were considered statistically significant if a *p*-value of less than 0.05 was reached.

## 3. Results

### 3.1. Self-Assembly of Tumor Spheroid on Hydrogel with Physiological Stiffness

**Figure 1:**
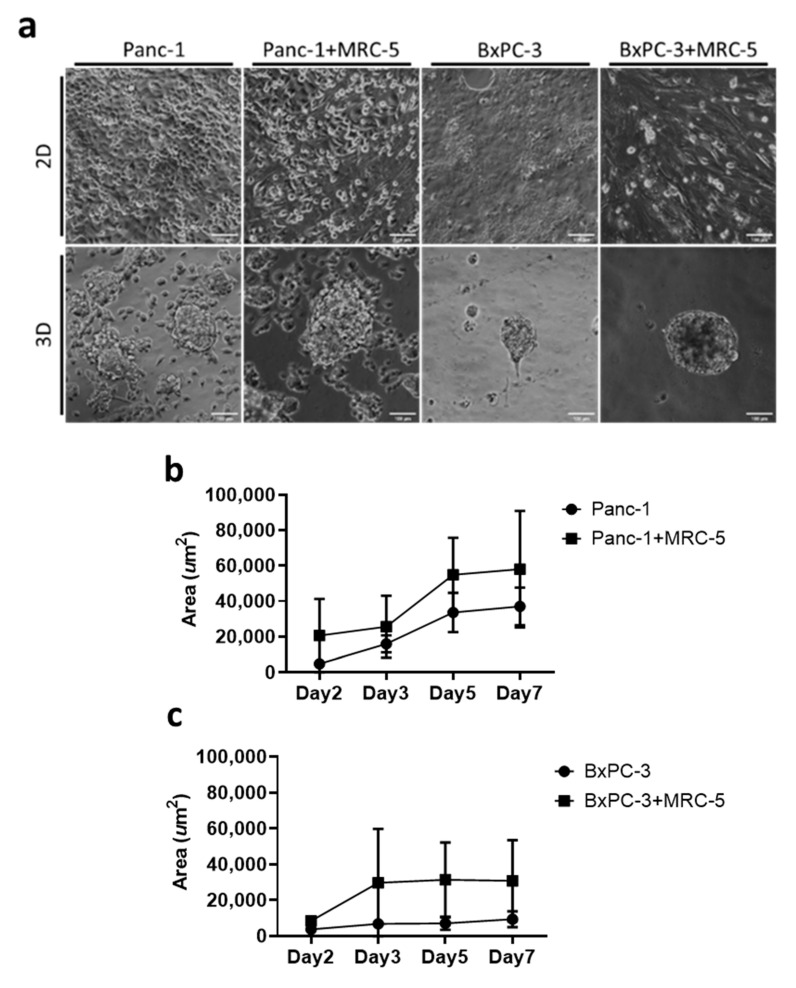
The formation of 3D tumoroids in an in vitro culture system. (**a**) Bright field images of pancreatic cancer cells Panc-1 and BxPC-3 with and without MRC-5 for 7 days in 2D and 3D conditions. Scale bar = 100 μm. (20× Magnification) (**b**,**c**) The tumoroid area was quantified using ImageJ 1.53j software; means and standard deviations are presented for each sample (average ± SD, *n* = 10).

To reconstruct the in vitro 3D tumor architecture, we developed a culture system using the substrate of PA hydrogel. The stiffness of the PA hydrogel was tuned to 1 kPa to recapture the physiological rigidity of pancreatic tissue [28]. In contrast to the monolayer of cells on conventional 2D petri dishes, both Panc-1 and BxPC-3 cells formed 3D spheroids spontaneously. On day 7, the area of Panc-1 spheroids was approximately 37,000 μm^2^, and the area of BxPC-3 spheroids was approximately 9300 μm^2^ (Figure 1a). Fibroblast cells (MRC-5) were used to mimic stroma cells in the microenvironment. In the presence of fibroblast cells, we observed an increased activity in the spheroid assembling process, in which the spheroid rapidly formed within 48 h. The size of spheroids of cancer cells and cocultured cells also exhibited a notable difference; the area of cocultured spheroids for both Panc-1 and BxPC-3 increased by approximately 0.5-fold and 2.5-fold, respectively, relative to cancer cells alone on day 7 (Figure 1b,c). With relatively little equipment, we demonstrated that the developed PA hydrogel substrate can be applied for 3D pancreatic spheroid culturing, with abundant cell–cell contact and a rich microenvironment [29]. Moreover, our data indicated that the surrounding fibroblast cells effectively modulated the intercellular interactions of pancreatic cells and promoted the assembly of both Panc-1 and BxPC-3 cells.

### 3.2. Biological Effectiveness of BNCT on the Pancreatic Tumor Spheroids

**Figure 2:**
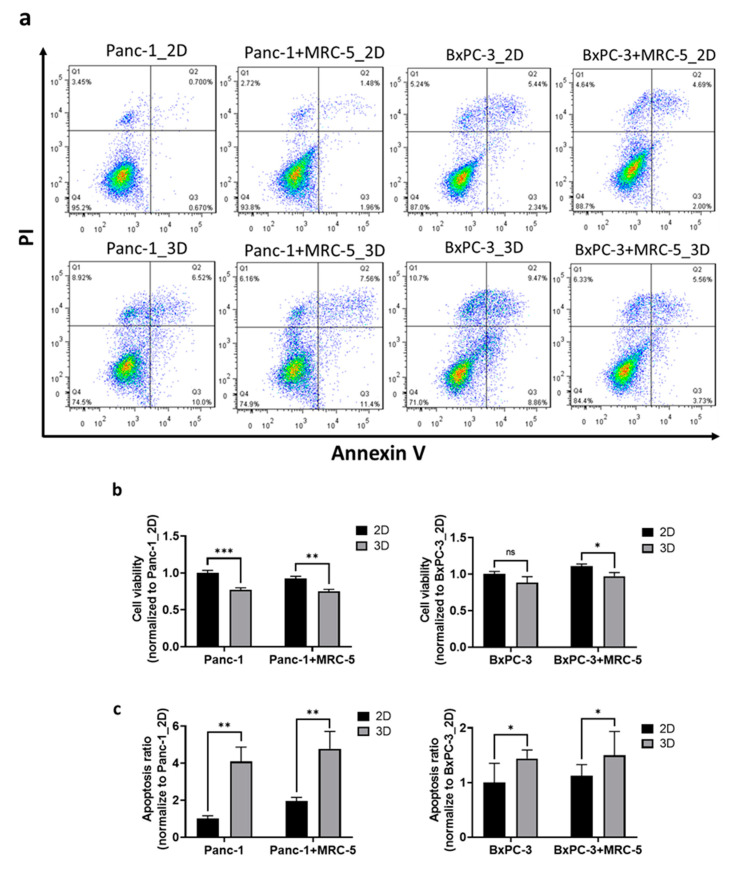
The pancreatic cancer cell expressed obvious BNCT susceptibility in an in vitro 3D culture system. (**a**) The density plot of flow cytometry results collected from cells stained with apoptosis marker annexin V (FITC) and nucleus marker propidium iodide (PI). *X*-axis and *Y*-axis represent the intensity of FITC-annexin V and PI, respectively. Each dot presents a cell and is allocated at the coordinate according to its relative fluorescent intensity level. Pseudo-color was added to show the population distribution. The quantitative results of (**b**) cell viability and (**c**) apoptosis in Panc-1 and BxPC-3 normalized to their 2D monolayer groups. Statistical significance was determined by *t*-test. ns, non-significant; * *p* < 0.05, ** *p* < 0.01, *** *p* < 0.001.

BNCT is a promising approach for pancreatic tumor treatment [30], but its biological effectiveness against pancreatic tumors is underexplored. We used 3D pancreatic tumor spheroids to evaluate the biological effectiveness of BNCT with BPA as the boron delivery reagent (Figure 2a). After thermal neutron irradiation, cancer cells in the spheroids exhibited lower survival rates (Panc-1: 69.7%, BxPC-3: 64.9%) than those in the 2D monolayers (Panc-1: 90.3%, BxPC-3: 73.5%); the presence of fibroblast cells had no significant influence on cell survival in our experiments (Figure 2b). Induced apoptosis is an essential mechanism driving the radio-therapeutic effects [30]. For Panc-1 cells, the results of an apoptosis assay indicated significantly increased apoptosis by approximately four times in the spheroids than in the 2D monolayers. The increased apoptotic response was also observed in the presence of fibroblast cells (Figure 2c). Similarly, a higher apoptotic response of BxPC-3 was noted in 3D spheroids than in 2D monolayers, although the increase was only 44% higher than in 2D cells. These findings suggest that the pancreatic cancer cells in 3D spheroids were more sensitive to the BNCT treatment than those in the conventional 2D culture; the fibroblast cells, in our experimental conditions, had no influence on cell viability or apoptosis. On the basis of the cell survival rate and the induced apoptosis, we als noted that Panc-3 cells were more sensitive to the alteration of architecture than BxPC-3 cells.

### 3.3. The BNCT Related Boron Uptake and Intercellular Contacts in Tumor Spheroids

**Figure 3:**
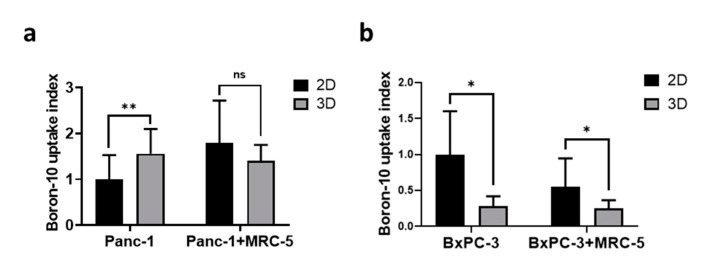
The monolayer culture exhibited higher boronophenylalanine (BPA) uptake than that of the 3D tumoroid model. The relative BPA uptake in (**a**) Panc-1 and (**b**) BxPC-3 spheroids and monolayer cells. The BPA uptake was normalized to 2D culture groups. Statistical significance was determined by *t*-test. ns, non-significant; * *p* < 0.05, ** *p* < 0.01.

The spatial organization of cells can affect the cellular microenvironment and various aspects of cellular physiology [31], which are known to be highly associated with the RBE of radiation therapies [32]. Because the intracellular distribution of ^10^B within tumors influences the RBE of BNCT treatment [33], we examined the difference in BPA uptake between 3D spheroids and 2D monolayers by using ICP-MS. For the Panc-1 cells, higher BPA uptake was observed in the 3D spheroids than in the 2D monolayers; no significant difference was observed between 2D and 3D Panc-1 culture conditions in the presence of fibroblast (Figure 3a). For BxPC-3 cells, lower BPA uptake (by approximately 40%) was observed in 3D spheroids, and the presence of fibroblast further reduced BPA uptake (Figure 3b).

**Figure 4:**
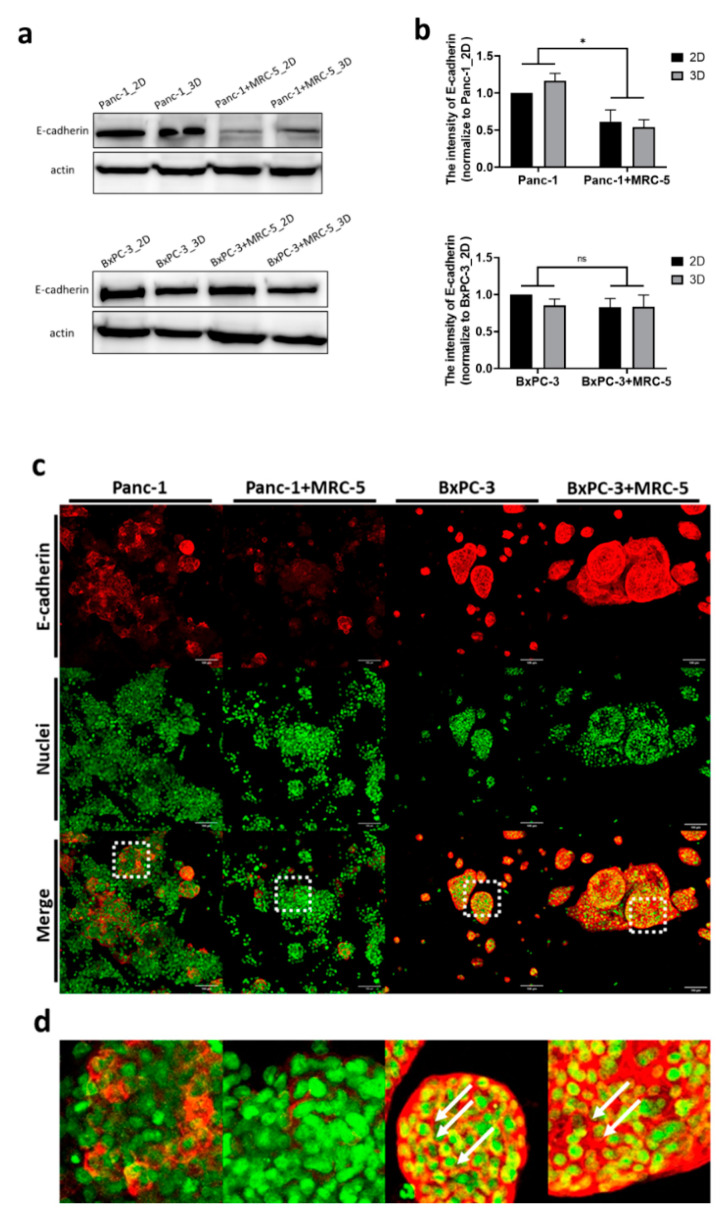
The expression of E-cadherin was upregulated in the 3D tumoroid structures and downregulated in Panc-1 when tumoroids were cocultured with fibroblasts. (**a**) The expression of E-cadherin determined by western blot assay and (**b**) the intensity quantified by ImageJ 1.53j software. (**c**) Immunofluorescence stained images of E-cadherin and Sytox (nuclei) in Panc-1 and BxPC-3 cancer cells grown alone or cocultured with MRC-5 after 7 days. The scale bar represents 100 μm. (20× Magnification) (**d**) The magnified confocal images of pancreatic tumoroids from (**c**). Statistical significance was determined by *t*-test. ns, non-significant; * *p* < 0.05.

In addition to the radiation dose, intercellular signal transduction serves a key role in regulating the cellular response to radiation [32]. E-cadherin is an indispensable protein of adherens junction for intercellular organization and tissue homeostasis, and its loss contributes to tumor metastasis and therapeutic resistance [34,35]. We compared the expression of E-cadherin in 3D tumor spheroids and 2D monolayers, and the results showed that E-cadherin was slightly increased (by 15%) in Panc-1 3D tumoroids although there was no such difference between BxPC-3 2D and 3D cultures. Notably, the quantitative results indicated that the amount of E-cadherin was dramatically downregulated (by ~50%) in Panc-1 cocultured with fibroblasts, but in BxPC-3, the E-cadherin was not affected (Figure 4a,b).

In addition to fibroblasts, the 3D microenvironment influences the signaling pathways of cancer cells [36]. Next, we investigated the spatial distribution of E-cadherin in our 3D culture system. Immunofluorescence staining was applied to study the expression of E-cadherin (Figure 4c). Using laser scanning confocal microscopy, we noticed the heterogeneous expression of E-cadherin across the 3D tumor spheroid axis. Obviously, the expression pattern of E-cadherin in 3D culture was cell type dependent. In Panc-1 cells, the E-cadherin was only expressed on certain cell surface and there was a weaker signal when cocultured with fibroblast. However, BxPC-3 cells had abundant expression of E-cadherin and appeared as a networked pattern in the entire tumoroid (Figure 4d). These findings suggest that, compared to BxPC-3, E-cadherin expression in Panc-1 tumoroid was dramatically influenced by fibroblasts and its distribution is also cell line dependent.

### 3.4. Clusters of Damaged Cells in 3D Pancreatic Tumor Spheroids after BNCT

**Figure 5:**
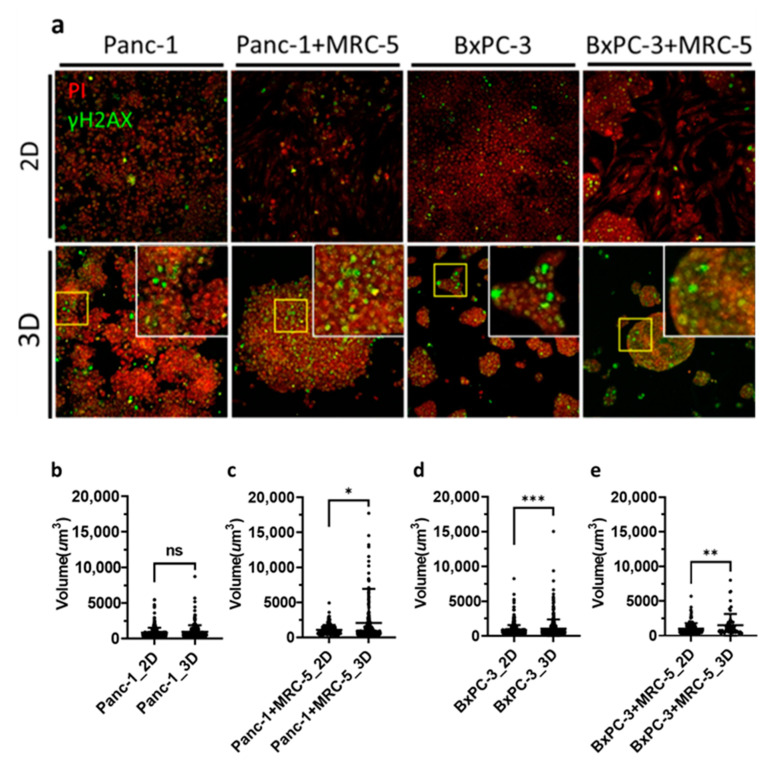
Immunofluorescence staining against phosphorylated histone H2AX (γH2AX) for BNCT-induced double strand break in pancreatic cancer cells. (**a**) Panc-1 and BxPC-3 cells treated with BPA before BNCT irradiation. Cells were fixed after irradiation and stained with γH2AX and propidium iodide. Images were obtained through confocal microscopy (20× Magnification). (**b**–**e**) Density dot plots of the volume of γH2AX foci; the mean values for 2D and 3D samples were (**b**) 865.3 and 971.9, (c) 1057 and 2053, (**d**) 857.8 and 1079, and (**e**) 1032 and 1515, respectively. ns, non-significant; * *p* < 0.05, ** *p* < 0.01, *** *p* < 0.001.

DNA double-strand breaks (DSBs) are highly correlated with radiation induced apoptosis and are widely used in RBE evaluation [37]. Here, we used phosphorylated H2AX (γH2AX) staining for the assessment of DSBs after irradiation [38]. Our data showed that after BNCT treatment, γH2AX was expressed as foci at the DSB sites in the nuclei in both 2D monolayers and 3D spheroids. In both Panc-1 and BxPC-3 cells, a unique pattern with DSB clusters was noted (Figure 5a). We quantified the number of cell clusters expressing γH2AX by measurement of the fluorescence volume (Figure 5b–e). In general, the results showed 1.5 to 2-fold higher foci volume in 3D spheroids than 2D monolayers, and there was no obvious impact of fibroblasts on the observed DSBs.

**Figure 6:**
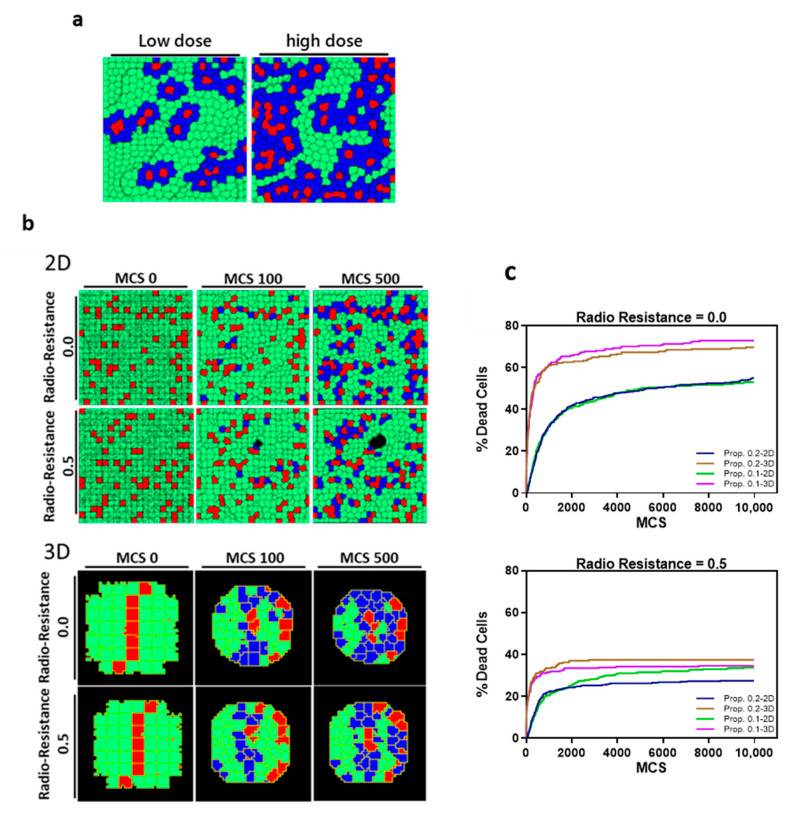
The predicted probability of BNCT treatment outcome through Monte Carlo simulation. (**a**) Proposed dose definitions in 2D and 3D model simulations with live tumor cells (green), dead cells (blue) and BPA-containing cells (red). (**b**) Representative images at successive Monte Carlo Timesteps (MCS) of cell death in 2D and 3D models post irradiation with high dose. (**c**) Cell death count in 2D sheets and 3D tumoroids of pancreatic tumor cells after BNCT treatment with different radioresistance thresholds (0.0 and 0.5) at varying particle propagation constants (0.1 and 0.2 pixel^2^/MCS).

A Monte Carlo simulation model was established to evaluate the influence of tumor architecture on DSBs caused by BNCT. The BNCT-induced DSBs were simulated under high and low drug dose conditions. The effective dose was used to present the percentage of cells which yield alpha particle after BNCT in the whole cell population. A high dose was presented as 15% and 5% was the low dose, and the initial spatial distribution of alpha particle releasing cells is random (Figure 6a). In both 2D and 3D models, we observed a clear dependence of cell viability on the effective dose of alpha particles administered. The high dose condition was used for the rest of the simulations. A radioresistance threshold was introduced to incorporate the different susceptibility of cells to BNCT, which is caused by different inert properties of cells. Alpha particle release was triggered at the beginning of the simulation; once the particle count within a cell reaches the pre-defined resistance threshold, it leads to cell death. In the 3D structure, the cell death count is substantially higher as compared to the 2D conditions (Figure 6b,c). The 3D tumoroid reaches a maxima death count of 60% at the high dose low threshold condition irrespective of the heavy particle propagation coefficient. This result shows a decrease when the tumor cells are grown in a monolayer. Even under an increased therapy resistance threshold, the 3D structure encounters a greater decrease in tumor cell count at 40% compared to the 30% in mono-sheet structure. The increased number of neighboring cells in 3D organization ensures that for the same dose level, a greater number of cells fall within the radiation zone as compared to the 2D planar layout. The propagation distance of heavy particles, characterized by the decay constant, determines the extent of radiation damage, whereas the propagation rate defined by the propagation coefficient seems to have little effect on the elimination of tumor cells by BNCT.

## 4. Discussion

With recent advancements in biomedical technology, 3D culture systems are applied to imitate complex cell–cell interaction and microenvironments in vivo [22]. Since it can recapture the complex multiple cellular organization and microenvironment in vivo, 3D culturing has been broadly recognized to provide more reliable estimations for cancer therapy compared with conventional 2D culturing [39]. For example, through cultivating cancer cells collected from patients in matrigel, the grown tumor spheroids, which represent tumor properties, were used by researchers for drug screening for personalized cancer treatment [40]. Instead of using matrigel matrix, we developed the 3D tumor spheroids with ECM-coated PA hydrogel. We found that the Panc-1 cells migrated aggressively and formed irregular colonies. On the other hand, BxPC-3 formed small clusters unlike the Panc-1 pattern. In addition to inducing the 3D architecture formation, our self-assembly method is adaptable when estimating the influence of stroma cells. Coculturing with fibroblasts, which is known to stimulate pancreatic tumor growth [41,42], both the pancreatic cancer cell lines in our system showed rapid assembling dynamics and grew into larger spheroids. Moreover, when cocultured with fibroblasts, the expression of E-cadherin was substantially decreased. The decrease of E-cadherin implied that the epithelial-mesenchymal transition of pancreatic tumors can be induced by stromal fibroblast cells [43], which is consistent with metastatic tumors in clinical reports [44]. Using the developed self-assembly culture platform, we can remodel certain physiological characters of pancreatic tumors and the surrounding matrix. In contrast to the current 3D culture platform, the assembled tumoroids lie on the same image plane spontaneously, which greatly facilitates the subsequent imaging process; without using any animal-derived substrates, our system is expected to reproduce specimens in a more controllable manner [45].

A large number of studies have demonstrated the impact of tissue architecture on radiation therapy [46,47]. For instance, Storch et al. demonstrated that the survival rate of human lung carcinoma cells was higher in a 3D aggregation culture compared with monolayer cell culture after γ-ray exposure [48]. Görte et al. revealed a higher RBE of protons compared to photons in a 3D pancreatic cancer model [49]. The three-dimensional architecture not only provides physical shielding for cells from radiation exposure, but also modifies the cell physiology. In 3D glioblastoma, the upregulated Akt is found, which can facilitate DNA repair by nonhomologous end-joining and homologous recombination [50]. Moreover, the integral membrane protein Cav-1, which induced Akt phosphorylation and β1 integrin upregulation, can promote the radioresistance in 3D pancreatic cancer models [51]. On the basis of these findings, higher radiation resistance is expected in 3D conditions rather than in 2D conditions. Notably, in contrast to most previous findings, our results exhibited that the 3D spheroids were less resistant to BNCT; lower survival rates and higher apoptosis were observed in the 3D pancreatic spheroids.

In order to interpret this unexpected finding, we first examined the intracellular boron concentration. Different concentrations of intracellular ^10^B, the neutron capture target to initiate the high LET particle therapy [16], were observed under different experimental conditions. For smaller spheroids of BxPC-3, the lower intracellular BPA indicated that the 3D structure seemed to constrain the free diffusion of BPA, rather than 2D monolayers (Figure 3b). In contrast, for Panc-1 cells, the drug concentration retained in the large 3D tumoroids seemed to be close to the one in 2D culture (Figure 3a). Thus, our results showed that, more than diffusion limitation, the intracellular BPA concentration may be affected by other factors, such as alteration of endocytosis in different cells, which further influence the therapeutic efficacy of BNCT.

The abundant cell–cell contact in 3D multicellular organization is thought to be associated with higher therapeutic resistance. The expression of cell adhesion molecules, such as E-cadherin or integrins, can promote cell survival signals and mediate radioresistance [52]. Under our experimental conditions, the Western blot results showed no significant difference in E-cadherin expression between 2D monolayers and 3D spheroids. The cocultured fibroblasts substantially downregulated the E-cadherin expression in Panc-1, but not in BxPC-3. Moreover, fluorescence microscopy revealed the notable difference in the distribution of E-cadherin between the pancreatic tumor spheroids. Within Panc-1 spheroids, no obvious E-cadherin was observed at the cell–cell contact, which implied weak intercellular adhesion and low diffusion barriers [53]. This partially explains the comparable BPA uptake by Panc-1 in 2D monolayers and 3D spheroids. For BxPC-3, we speculate that the vivid E-cadherin signal seen aligned with cell contacts not only relates to robust diffusion constraint [54], but also associates with the high therapeutic resistance of BxPC-3 [55]. In summary, our results suggest that cell–cell contact may partially influence cellular viability but was not the major determining factor behind the low BNCT resistance of 3D spheroids in our experiment.

After a comparison of the apoptotic response of Panc-1 and BxPC-3 cells, we speculated that stronger E-cadherin expression and lower intercellular boron concentration could result in heightened BNCT resistance. We also noted that substantial apoptosis was observed in the 3D tumoroids compared with those in the 2D culture. Our results indicated that cells exhibited higher susceptibility to BNCT while growing as 3D spheroids than as 2D monolayers.

Here, we proposed a hypothetical model wherein gamma rays and alpha particles released from ^10^B-uptaking cells caused DNA DSBs and resulted in apoptotic responses and subsequent cell death. This effect can influence neighboring cells to cause field destruction in 3D structures. As in the 3D physical particle contour reported in a previous BNCT study [56], cells within a spheroid structure are damaged by particles omnidirectionally, whereas the 2D monolayer structure dramatically reduces the probability of alpha particle exposure. DSBs and MC simulation supported our hypothetical model; clustered cells with γ-H2AX foci suggested that the cell damage was limited within small energy transfer region with higher exposure probability. Moreover, we used the volume of γ-H2AX signal to evaluate the injury zone, and the quantitative results indicated a larger damage contour inside the 3D tumoroid than the 2D monolayer. Moreover, high BNCT sensation of the 3D spheroid showed in MC simulation consisted with the observation of DSBs and further supported our hypothetical model. In contrast to the DSBs and apoptosis, the minor change in 2D and 3D survival rates implied a complex impact of multicellular organization [57]. For instance, other microenvironmental factors, such as the gradients of oxygen concentration to build a hypoxic environment in the spheroid core and HIF-1α signaling expressed in hypoxia, are also involved in the sensitization to radiation treatments [58,59]. Further studies are needed to explore the underlying mechanism in detail.

Though studies support the potential application of BNCT for pancreatic cancer treatment [60], the concerns regarding the damage to the intra-abdominal organs still exist and further constrains its clinical application [61]. With consideration of the rapid progress in boron agent development and neutron sources improvement, such as accelerator-based neutron source and Void collimation [62], BNCT is expected to be one of the promising radiotherapies for pancreatic cancer in the near future.

## 5. Conclusions

In this study, we developed a 3D pancreatic in vitro model by using cell assembly, which recaptured the cell–contact signaling and diffusion barriers for studies of BNCT. The unexpected low BNCT resistance of 3D spheroids suggested the unique biological effectiveness of BNCT relative to other radiotherapies. In our experiment, multicellular structures altered the exposure probability of cells to α-particles, which was highly associated with DSBs and apoptosis. An MC simulation was applied to estimate the drug uptake and physiological responses for further evaluating BNCT efficacy. Our results on the effectiveness of BNCT at cellular levels can contribute to better understanding of radiobiology, and the developed in vitro model and simulation tools may facilitate the development of personalized radiotherapy.

## Data Availability

The data generated during the current study are available from the corresponding authors upon reasonable request.

## References

[1] Rawla P., Sunkara T., Gaduputi V. (2019). Epidemiology of Pancreatic Cancer: Global Trends, Etiology and Risk Factors. World J. Oncol..

[2] Ryan D.P., Hong T.S., Bardeesy N. (2014). Pancreatic Adenocarcinoma. N. Engl. J. Med..

[3] NIH Pancreatic Cancer Treatment (PDQ®) Patient Version. https://www.cancer.gov.

[4] Cancer.Net (2020). Pancreatic Cancer: Statistics. https://www.cancer.net.

[5] Gastrointestinal Tumor Study Group (1987). Further evidence of effective adjuvant combined radiation and chemotherapy following curative resection of pancreatic cancer. Cancer.

[6] Kalser M.H., Ellenberg S.S. (1985). Pancreatic cancer. Adjuvant combined radiation and chemotherapy following curative resection. Arch. Surg..

[7] Neoptolemos J.P., Stocken D.D., Friess H., Bassi C., Dunn J.A., Hickey H., Beger H., Fernandez-Cruz L., Dervenis C., Lacaine F. (2004). A Randomized Trial of Chemoradiotherapy and Chemotherapy after Resection of Pancreatic Cancer. N. Engl. J. Med..

[8] Moding E.J., Kastan M.B., Kirsch D.G. (2013). Strategies for optimizing the response of cancer and normal tissues to radiation. Nat. Rev. Drug Discov..

[9] Barth R.F., Coderre J.A., Vicente M.G.H., Blue T.E. (2005). Boron neutron capture therapy of cancer: Current status and future prospects. Clin. Cancer Res..

[10] Kawabata S., Suzuki M., Hirose K., Tanaka H., Kato T., Goto H., Narita Y., Miyatake S.-I. (2021). Accelerator-based BNCT for patients with recurrent glioblastoma: A multicenter phase II study. Neuro-Oncol. Adv..

[11] Suzuki M. (2020). Boron neutron capture therapy (BNCT): A unique role in radiotherapy with a view to entering the accelerator-based BNCT era. Int. J. Clin. Oncol..

[12] Barker H.E., Paget J.T.E., Khan A.A., Harrington K.J. (2015). The tumour microenvironment after radiotherapy: Mechanisms of resistance and recurrence. Nat. Rev. Cancer.

[13] Vaupel P. (2004). Tumor microenvironmental physiology and its implications for radiation oncology. Semin. Radiat. Oncol..

[14] Bissell M.J., Hines W.C. (2011). Why don’t we get more cancer? A proposed role of the microenvironment in restraining cancer progression. Nat. Med..

[15] Krisnawan V.E., Stanley J.A., Schwarz J.K., DeNardo D.G. (2020). Tumor Microenvironment as a Regulator of Radiation Therapy: New Insights into Stromal-Mediated Radioresistance. Cancers.

[16] Moss R.L. (2014). Critical review, with an optimistic outlook, on Boron Neutron Capture Therapy (BNCT). Appl. Radiat. Isot..

[17] Moon H.-r., Han B., Park K. (2020). 15—Engineered tumor models for cancer biology and treatment. Biomaterials for Cancer Therapeutics.

[18] Sørensen B.S., Bassler N., Nielsen S., Horsman M.R., Grzanka L., Spejlborg H., Swakoń J., Olko P., Overgaard J. (2017). Relative biological effectiveness (RBE) and distal edge effects of proton radiation on early damage in vivo. Acta Oncol..

[19] Mak I.W., Evaniew N., Ghert M. (2014). Lost in translation: Animal models and clinical trials in cancer treatment. Am. J. Transl. Res..

[20] Breslin S., O’Driscoll L. (2013). Three-dimensional cell culture: The missing link in drug discovery. Drug Discov. Today.

[21] Trédan O., Galmarini C.M., Patel K., Tannock I.F. (2007). Drug Resistance and the Solid Tumor Microenvironment. JNCI J. Natl. Cancer Inst..

[22] Kapałczyńska M., Kolenda T., Przybyła W., Zajączkowska M., Teresiak A., Filas V., Ibbs M., Bliźniak R., Łuczewski Ł., Lamperska K. (2018). 2D and 3D cell cultures—a comparison of different types of cancer cell cultures. Arch. Med. Sci..

[23] Andasari V., Roper R.T., Swat M.H., Chaplain M.A.J. (2012). Integrating Intracellular Dynamics Using CompuCell3D and Bionetsolver: Applications to Multiscale Modelling of Cancer Cell Growth and Invasion. PLoS ONE.

[24] Swat M.H., Thomas G.L., Belmonte J.M., Shirinifard A., Hmeljak D., Glazier J.A. (2012). Multi-scale modeling of tissues using CompuCell3D. Methods Cell Biol..

[25] Glazier J.A., Graner F. (1993). Simulation of the differential adhesion driven rearrangement of biological cells. Phys. Rev. E.

[26] Graner F., Glazier J.A. (1992). Simulation of biological cell sorting using a two-dimensional extended Potts model. Phys. Rev. Lett..

[27] Tse J.R., Engler A.J. (2010). Preparation of Hydrogel Substrates with Tunable Mechanical Properties. Curr. Protoc. Cell Biol..

[28] Rice A.J., Cortes E., Lachowski D., Cheung B.C.H., Karim S.A., Morton J.P., Del Río Hernández A. (2017). Matrix stiffness induces epithelial—mesenchymal transition and promotes chemoresistance in pancreatic cancer cells. Oncogenesis.

[29] Tomás-Bort E., Kieler M., Sharma S., Candido J.B., Loessner D. (2020). 3D approaches to model the tumor microenvironment of pancreatic cancer. Theranostics.

[30] Balcer-Kubiczek E.K. (2012). Apoptosis in Radiation Therapy: A Double-Edged Sword. Exp. Oncol..

[31] Kojima M., Higuchi Y., Yokota M., Ishii G., Saito N., Aoyagi K., Sasaki H., Ochiai A. (2014). Human Subperitoneal Fibroblast and Cancer Cell Interaction Creates Microenvironment That Enhances Tumor Progression and Metastasis. PLoS ONE.

[32] Ashrafizadeh M., Farhood B., Eleojo Musa A., Taeb S., Najafi M. (2020). The interactions and communications in tumor resistance to radiotherapy: Therapy perspectives. Int. Immunopharmacol..

[33] Barth R.F., Mi P., Yang W. (2018). Boron delivery agents for neutron capture therapy of cancer. Cancer Commun..

[34] Alimperti S., Andreadis S.T. (2015). CDH2 and CDH11 act as regulators of stem cell fate decisions. Stem Cell Res..

[35] Graziano F., Corso G., Roviello F. (2013). The E-Cadherin Gene, Structure and Function. Spotlight on Familial and Hereditary Gastric Cancer.

[36] Davies A.E., Albeck J.G. (2018). Microenvironmental Signals and Biochemical Information Processing: Cooperative Determinants of Intratumoral Plasticity and Heterogeneity. Front. Cell Dev. Biol..

[37] Kaina B. (2003). DNA damage-triggered apoptosis: Critical role of DNA repair, double-strand breaks, cell proliferation and signaling. Biochem. Pharmacol..

[38] Firsanov D.V., Solovjeva L.V., Svetlova M.P. (2011). H2AX phosphorylation at the sites of DNA double-strand breaks in cultivated mammalian cells and tissues. Clin. Epigenetics.

[39] Stadler M., Walter S., Walzl A., Kramer N., Unger C., Scherzer M., Unterleuthner D., Hengstschläger M., Krupitza G., Dolznig H. (2015). Increased complexity in carcinomas: Analyzing and modeling the interaction of human cancer cells with their microenvironment. Semin. Cancer Biol..

[40] Ravi M., Ramesh A., Pattabhi A. (2017). Contributions of 3D Cell Cultures for Cancer Research. J. Cell. Physiol..

[41] Bolm L., Cigolla S., Wittel U.A., Hopt U.T., Keck T., Rades D., Bronsert P., Wellner U.F. (2017). The Role of Fibroblasts in Pancreatic Cancer: Extracellular Matrix Versus Paracrine Factors. Transl. Oncol..

[42] Olumi A.F., Grossfeld G.D., Hayward S.W., Carroll P.R., Tlsty T.D., Cunha G.R. (1999). Carcinoma-associated Fibroblasts Direct Tumor Progression of Initiated Human Prostatic Epithelium. Breast Cancer Res..

[43] Loh C.-Y., Chai J.Y., Tang T.F., Wong W.F., Sethi G., Shanmugam M.K., Chong P.P., Looi C.Y.J.C. (2019). The E-cadherin and N-cadherin switch in epithelial-to-mesenchymal transition: Signaling, therapeutic implications, and challenges. Cells.

[44] Deer E.L., González-Hernández J., Coursen J.D., Shea J.E., Ngatia J., Scaife C.L., Firpo M.A., Mulvihill S.J. (2010). Phenotype and genotype of pancreatic cancer cell lines. Pancreas.

[45] Song H.H., Park K.M., Gerecht S. (2014). Hydrogels to model 3D in vitro microenvironment of tumor vascularization. Adv. Drug Deliv. Rev..

[46] Griffin R.J., Williams B.W., Park H.J., Song C.W. (2005). Preferential action of arsenic trioxide in solid-tumor microenvironment enhances radiation therapy. Int. J. Radiat. Oncol. Biol. Phys..

[47] Wishart G., Gupta P., Schettino G., Nisbet A., Velliou E. (2021). 3d tissue models as tools for radiotherapy screening for pancreatic cancer. Br. J. Radiol..

[48] Storch K., Eke I., Borgmann K., Krause M., Richter C., Becker K., Schröck E., Cordes N. (2010). Three-dimensional cell growth confers radioresistance by chromatin density modification. Cancer Res..

[49] Görte J., Beyreuther E., Danen E.H.J., Cordes N. (2020). Comparative Proton and Photon Irradiation Combined with Pharmacological Inhibitors in 3D Pancreatic Cancer Cultures. Cancers.

[50] Gomez-Roman N., Chong M.Y., Chahal S.K., Caragher S.P., Jackson M.R., Stevenson K.H., Dongre S.A., Chalmers A.J. (2020). Radiation Responses of 2D and 3D Glioblastoma Cells: A Novel, 3D-specific Radioprotective Role of VEGF/Akt Signaling through Functional Activation of NHEJ. Mol. Cancer Ther..

[51] Hehlgans S., Eke I., Storch K., Haase M., Baretton G.B., Cordes N. (2009). Caveolin-1 mediated radioresistance of 3D grown pancreatic cancer cells. Radiother. Oncol..

[52] Josson S., Sharp S., Sung S.-Y., Johnstone P.A.S., Aneja R., Wang R., Gururajan M., Turner T., Chung L.W.K., Yates C. (2010). Tumor-Stromal Interactions Influence Radiation Sensitivity in Epithelial- versus Mesenchymal-Like Prostate Cancer Cells. J. Oncol..

[53] van Roy F., Berx G. (2008). The cell-cell adhesion molecule E-cadherin. Cell. Mol. Life Sci..

[54] Zihni C., Mills C., Matter K., Balda M.S. (2016). Tight junctions: From simple barriers to multifunctional molecular gates. Nat. Rev. Mol. Cell Biol..

[55] Mazzeo E., Hehlgans S., Valentini V., Baumann M., Cordes N. (2012). The Impact of Cell-Cell Contact, E-Cadherin and EGF Receptor on the Cellular Radiosensitivity of A431 Cancer Cells. Radiat. Res..

[56] D’Anselmi F., Masiello M.G., Cucina A., Proietti S., Dinicola S., Pasqualato A., Ricci G., Dobrowolny G., Catizone A., Palombo A. (2013). Microenvironment Promotes Tumor Cell Reprogramming in Human Breast Cancer Cell Lines. PLoS ONE.

[57] Shuryak I. (2019). Review of resistance to chronic ionizing radiation exposure under environmental conditions in multicellular organisms. J. Environ. Radioact..

[58] Du J., Sun B., Zhao X., Gu Q., Dong X., Mo J., Sun T., Wang J., Sun R., Liu Y. (2014). Hypoxia promotes vasculogenic mimicry formation by inducing epithelial–mesenchymal transition in ovarian carcinoma. Gynecol. Oncol..

[59] Liu Y., Mohri Z., Alsheikh W., Cheema U. (2021). The Role of Biomimetic Hypoxia on Cancer Cell Behaviour in 3D Models: A Systematic Review. Cancers.

[60] Chou F.I., Chung H.P., Liu H.M., Chi C.W., Lui W.Y. (2009). Suitability of boron carriers for BNCT: Accumulation of boron in malignant and normal liver cells after treatment with BPA, BSH and BA. Appl. Radiat. Isot..

[61] Gavin P.R., Kraft S.L., Dehaan C.E., Swartz C.D., Griebenow M.L. (1994). Large animal normal tissue tolerance with boron neutron capture. Int. J. Radiat. Oncol. Biol. Phys..

[62] Yanagie H., Sakurai Y., Ogura K., Kobayashi T., Furuya Y., Sugiyama H., Kobayashi H., Ono K., Nakagawa K., Takahashi H. (2007). Evaluation of neutron dosimetry on pancreatic cancer phantom model for application of intraoperative boron neutron-capture therapy. Biomed. Pharmacother..

